# Venous dynamics in anesthetized sheep govern postural‐induced changes in cerebrospinal fluid pressure comparable to those in humans

**DOI:** 10.14814/phy2.15525

**Published:** 2022-12-21

**Authors:** Nina Eva Trimmel, Anthony Podgoršak, Markus Florian Oertel, Simone Jucker, Margarete Arras, Marianne Schmid Daners, Miriam Weisskopf

**Affiliations:** ^1^ Center for Surgical Research University Hospital Zurich, University of Zurich Zurich Switzerland; ^2^ Department of Mechanical and Process Engineering, ETH Zurich Zurich Switzerland; ^3^ Department of Neurosurgery University Hospital Zurich, University of Zurich Zurich Switzerland

**Keywords:** cerebrospinal fluid dynamics, jugular collapse, jugular venous pressure, postural change, sheep

## Abstract

Sheep are popular large animals in which to model human disorders and to study physiological processes such as cerebrospinal fluid dynamics. However, little is known about vascular compensatory mechanisms affecting cerebrospinal fluid pressures during acute postural changes in sheep. Six female white Alpine sheep were anesthetized to investigate the interactions of the vascular and cerebrospinal fluid system by acquiring measurements of intracranial pressure and central and jugular venous pressure during passive postural changes induced by a tilt table. The cross‐sectional area of the common jugular vein and venous blood flow velocity was recorded. Anesthetized sheep showed bi‐phasic effects of postural changes on intracranial pressure during tilting. A marked collapse of the jugular vein was observed during head‐over‐body tilting; this is in accordance with findings in humans. Active regulatory effects of the arterial system on maintaining cerebral perfusion pressure were observed independent of tilting direction. Conclusion: Anesthetized sheep show venous dynamics in response to posture‐induced changes in intracranial pressure that are comparable with those in humans.

## Graphical abstract

Posture‐induced changes in the venous system are well described in humans to control cerebrospinal fluid pressure in the upright position. This study aimed to examine this physiologic behavior in a quadruped species (sheep), which in addition, only has a common jugular vein, compared to humans which have an internal and an external jugular vein.

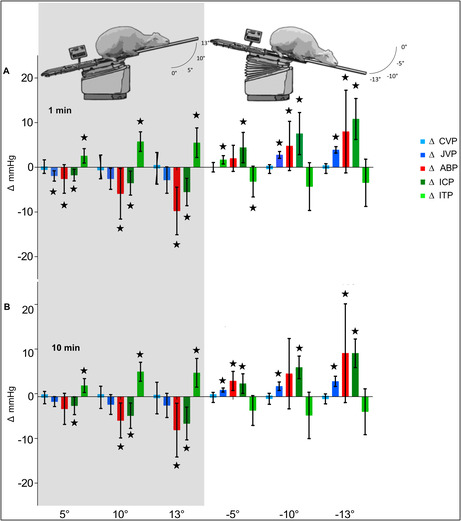

## 1 INTRODUCTION

Intracranial pressure (ICP), i.e., the pressure inside the skull, comprises arterial, venous, and cerebrospinal fluid (CSF) pressures (Kasprowicz et al., 2016). Davson's equation describes ICP and its dependency on CSF outflow resistance (*R*_out_), CSF production, and sagittal sinus pressure (SSP) (Davson et al., 1970). Aside from intracranial factors, extracranial factors are known to play a role in the maintenance of ICP (O'Connell, 1943). In particular, changes in the systemic arterial and venous system have shown to have a marked effect on ICP. CSF pulse wave dependency on the cardiac beat has been studied since the mid‐1950 s (Bering Jr., 1955; Dardenne et al., 1969) and has shown to be mainly arterial in origin (Kasuga et al., 1987; Sibayan et al., 1970) with slow rhythmic waves reflecting the SSP and central venous pressure (CVP) (Hamer et al., 1977) as well as respiratory waveforms associated with the respiratory cycle (Podgoršak et al., 2022). An inverse relationship between arterial blood pressure (ABP) and CSF pulse pressure was found in anesthetized dogs, where an increase in CSF pulse pressure was observed during arterial hypotension and the reverse occurred during arterial hypertension (Avezaat et al., 1980). An insufficiency of the right atrium, with a consecutive increase in right atrial pressure and CVP, has been shown to simultaneously increase mean CSF pressure in a canine model (Hamer et al., 1977), emphasizing the role of the pressure in the venous system in adequate CSF absorption. Further interaction and interdependence of the various compartments was previously shown in a porcine model where the effect of an increased intra‐abdominal pressure on ICP, mediated by an altered CVP due to mechanical constriction of the inferior vena cava, was demonstrated (Rosenthal et al., 1998).

Posture has been described to have a marked effect on CSF pressure and all the adjacent compartments thereof (Ballambat et al., 2022; Cresswell et al., 2012; Qvarlander et al., 2013; Rosenthal et al., 1998). The influence of postural changes in patients on CSF pressure in general, and of posture‐independent CSF pressure level, hydrostatic indifferent point, and the derived posture‐related intracranial‐to‐spinal compliance shift have been described previously (Magnaes, 1976a, 1976b; Magnaes, 1978; Raabe et al., 1999). Gehlen et al. (2017) attributed the observed craniospinal volume and compliance shift in humans to the collapse of the internal jugular vein which occurs during changes in posture from a horizontal to an upright position. Qvarlander et al. (2013) further investigated posture‐dependent jugular vein patency and, using three analytical models, validated ICP measurements at various tilt angles in normal pressure hydrocephalus patients. According to numerous studies, in the head over body (HoB) position, ICP decreases rapidly and significantly (Davenport et al., 1990; Durward et al., 1983; Feldman et al., 1992; Schneider et al., 1993). There seem to be two primary pathways contributing to this ICP decrease. One is the increase in venous outflow (Potts & Deonarine, 1973, Magnaes, 1976a) and the other is hypothesized to result from hydrostatic CSF displacement into the spinal subarachnoid space (Kenning et al., 1981; Magnaes, 1978). The subsequent negative venous pressure detected in cervical veins that is induced by the collapse of the internal jugular vein (Holmlund et al., 2018) thereby counteracts the emergence of a pressure gradient between the intracranial CSF pressure and the SSP that would hamper CSF absorption in the upright position in humans.

This venous behavior appears to be true as well for other species, such as cats, dogs, and rodents, which, like humans, have an internal and external jugular vein (Kawajiri et al., 1983; Kotani, Momota, et al., 1992; Sakata et al., 1999). In cats, a 20° body over head (BoH) position leads to an increase in ICP, SSP, and mean ABP. As ICP and SSP increase proportionally, no changes in CSF absorption results (Kotani, Nitta, et al., 1992). Total occlusion of both internal jugular veins in all three species, however, is associated with a significant increase in ICP (Kawajiri et al., 1983; Kotani, Momota, et al., 1992; Sakata et al., 1999), underlining the hypothesis that absorption of the CSF into venous blood occurs mainly through the arachnoid villi into the dural venous sinuses, driven by the pressure gradient between CSF and SSP (Mortensen & Weed, 1934).

Sheep are popular large animals with which to model human neurological disorders, as their size and anatomy are comparable with those of humans, with high levels of structural homology (Murray & Mitchell, 2022; Trimmel et al., 2022). However, sheep, like other ruminants, have only a common jugular vein (JV) draining most of the blood from the head and neck (Lu et al., 2008; Hoffmann et al., 2014). Currently, to our knowledge, there is no description of jugular venous behavior during postural changes in sheep in the literature. In giraffes, which also only have a common JV, jugular venous blood pooling and a significant reduction in ABP and cardiac output is described when lowering the head below the body (Brondum et al., 2009). A JV collapse is described to occur when the head is brought back to an upright position.

We hypothesized that postural changes in sheep would show venous compensatory mechanisms to maintain intracranial CSF homeostasis similar to those of humans and other species. Hence, we investigated the interactions of the vascular and CSF systems in anesthetized sheep by acquiring measurements of ABP, CVP, jugular venous pressure (JVP), ICP, and intrathecal pressure (ITP) at the lumbar site during passive postural changes induced by a tilt table. In addition, the cross‐sectional area of the JV and venous blood flow velocity were assessed.

## 2 MATERIALS AND METHODS

### 2.1 Animals

Six female white Alpine sheep (*Ovis gmelini aries*) were included in this study. All sheep were mature (2–5 years old), with an average bodyweight of 75.6 ± 12.1 kg. After arrival at our facility, the sheep were kept in groups and were given a minimum of 1 week to acclimatize to their new surroundings. The health status of the animals was guaranteed by the conventional livestock supplier through regular screening and subsequent official attestation in accordance with the guidelines of the Swiss Federal Food Safety and Veterinary Office, as well as by a clinical examination conducted by an on‐site veterinarian. The animals had free access to hay and water and a mineral‐licking stone was provided. Room temperature was kept at 19 °C, with relative humidity ranging from 45% to 55%. All experimental procedures were conducted during the winter months in the northern hemisphere. Animal rooms were illuminated from 7:00 am to 5:00 pm, with a skylight to offer natural daylight. Each animal's overall health was assessed twice daily by an animal caretaker and on a regular basis by the veterinarian.

#### 2.1.1 Animal preparation and anesthesia

The sheep were fasted for 16–18 h prior to surgery. During fasting, unlimited access to water was guaranteed. On the day of the operation, a venous catheter (Braunüle®MT Luer Lock, 14 G, B. Braun Medical) was inserted into the sheep's left JV. The animal was pre‐medicated with 3 mg/kg body weight (BW) ketamine hydrochloride (Ketasol®100, Dr. E. Graeub AG) and 0.3 mg/kg BW midazolam (Dormicum® Roche Pharma) intravenously. Before orotracheal intubation, 2–5 mg/kg BW of propofol (Propofol‐®Lipuro 1%, B.Braun Medical AG) was administered intravenously. Under sterile conditions, a urinary catheter was placed transurethrally (Rüsch Gold 3way, 18 F, Teleflex Medical Enterprise). An orogastric tube (Rüsch 12 mm, Teleflex Medical Enterprise, Athlone, Ireland) was placed to drain excess gastric fluid and reduce ruminal gases. A naso‐esophageal temperature probe was introduced for continuous monitoring of body temperature. The sheep were positive‐pressure‐ventilated throughout the experiment (18 breaths/min, tidal volume 10–15 ml/kg BW, FiO_2_ 0.7, Pmax 30 mmHg). Inhalation of 1–2% isoflurane (Attane™ Isoflurane ad.us.vet., Piramal Enterpr. India) in an oxygen/air mixture was used to maintain anesthesia. Additionally, propofol was infused at a steady rate (3 mg/kg BW/h). Sufentanil (Sufenta®forte, Janssen‐Cilag AG, Zug, Switzerland; 2.5 g/kg BW/h i.v.) was administered for intraoperative analgesia. To maintain intraoperative fluid balance, Ringer's solution (Ringerfundin, B.Braun Medical AG) was infused at 5 ml/kg BW/h.

### 2.2 Instrumentation

**FIGURE 1:**
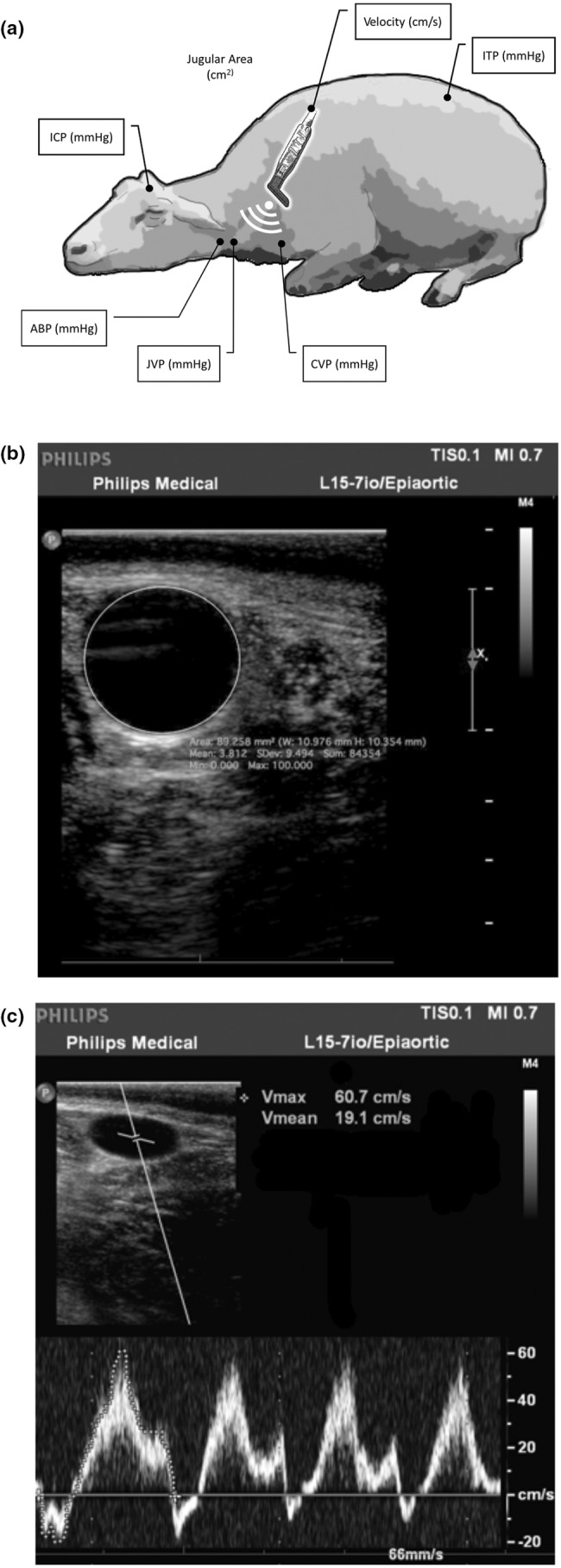
(a) Schematic depiction of anatomical sites of pressure sensor placements. ICP: intracranial pressure; ITP: intrathecal pressure; ABP: arterial blood pressure in the carotid artery; JVP: jugular venous pressure; CVP: central venous pressure in the anterior vena cava. (b) Representative ultrasonographic image of common jugular vein measurement of cross‐sectional area, width, and height. (c) Representative ultrasonographic image of pressure wave (PW) Doppler used to assess velocity and flow in the common jugular vein.

All pressures were measured by catheters placed percutaneously or surgically at the corresponding anatomical site (Figure 1a). The sheep were placed in sternal position throughout the experiment.

#### 2.2.1 Placement of intravascular catheters for pressure measurements

Under ultrasonographic guidance (iE33, Philips), intravascular catheters for continuous monitoring of carotid ABP, CVP, and JVP were inserted percutaneously by the Seldinger method. A 4F introducer sheath (Avanti®, Cordis®Corporation) was placed in the left carotid artery, a multilumen catheter (Arrow®Multilumen Central Venous BlueFlextip® Catheter, 20 cm in length, Arrow Int. Inc.) was inserted through the left JV into the anterior vena cava and a short 4F catheter (Avanti®, Cordis®Corporation) was inserted proximal to the CVP catheter into the JV.

#### 2.2.2 Implantation of intraventricular and intrathecal pressure probes

Using a diamond drill, a frontal burr hole (approximately 5 mm in diameter) trephination was performed approximately 2 cm anterior and 2 cm lateral to the posterior fontanelle at an angle of 10° from the sagittal plane. A 4.5F soft catheter (Neuromedex GmbH) was implanted into the right lateral cerebral ventricle; the positioning of the catheter was verified by CSF flow. To ensure accurate fixation and prevention of CSF leakage, a bone wax plug (Ethicon® Bone wax, Johnson & Johnson Medical Ltd.) was molded around the inserted catheter to cover the burr hole in the skull.

The intervertebral space between L6 and L7 was identified by lumbar spine X‐ray (Allura Xper FD 20, Philips Medical Systems Netherlands BV). After performing a laminotomy and minimal dural incision, a 4.5 F intrathecal catheter (Neuromedex GmbH) was inserted into the subarachnoid space. To prevent significant CSF leakage and blood from entering the intrathecal region, a hemostatic gauze strip (Tabotamp Ethicon, Johnson & Johnson Medical, Neuchatel, Switzerland) was placed over the opening in the dura. A bone wax plug was used around the exiting catheter to seal the bone defect, ensure adequate catheter fixation, and prevent CSF leakage.

### 2.3 Tilt test

The sheep were placed on a memory foam mattress on the surgical table. Tilting of the table was performed progressively in head over body (HoB) position at positive tilt angles of +5°, +10°, and +13° followed by the progressive body over head (BoH), position at negative tilt angles of −5°, −10°, and −13°. Each tilt angle was maintained for 10 min. Following the HoB position, the animal was stepwise brought back to a 0° position and a new baseline was acquired prior to starting the BoH tilting. ICP, ITP, ABP, JVP, and CVP were recorded continuously over all tilt angles. The mean of the recorded data for the first minute of the respective tilt angle and the mean of the 10th minute of the respective tilt angle was used for the analyses.

### 2.4 Pressure data acquisition

All catheters were connected to DTXPlus pressure transducers (Argon Medical Devices). To achieve hydrostatic equivalency, intracranial and intrathecal transducers were calibrated to ambient pressure at the level of the lateral cerebral ventricles. At the level of the right atrium, the arterial and central venous sensors were zeroed, while the jugular venous sensor was zeroed at the height of the tip of the jugular pressure catheter.

Using the commercially available software Ponemah v5.1 (Data Science International) and the ACQ‐7700 acquisition system, baseline pressure data were recorded during 10‐min with no disturbances. Data were acquired at a sample frequency of 1 kHz, then discriminated to 100 Hz.

### 2.5 Cross‐sectional area of the common JV and venous flow velocity

Height, width, and cross‐sectional area of the right JV were measured using a broadband linear transducer (L15‐7io/Epiaortic, Philips Medical Systems Nederland B.V), which was manually placed perpendicularly to the vessel (Figure 1b), approximately halfway between the head and the thoracic aperture. Aside from vascular dimensions, maximum and mean venous blood flow velocities were determined using angle‐corrected pulse wave (PW) Doppler mode focused on the center of the vein (Figure 1c). All measurements were performed at each given tilt angle at 1 and 10 min. Images were analyzed manually on an open‐source medical image viewer (Horos™, Horosproject.org).

### 2.6 Data analysis and statistics

Data were analyzed using custom Python 3.7.10‐written scripts (Open Source, Python Software Foundation). Mean pressures at baseline were determined as 5‐min arithmetic means, and data are given as mean ± standard deviation. Fast Fourier transform (FFT) was used to locate the cardiac and respiratory bands in the ABP baseline data. The heart rate of each sheep was determined as the peak of the cardiac band within the FFT spectrum. Microsoft Excel was used to compute each sheep's arithmetic means and standard deviations (Microsoft Corporation). Delta values for each parameter were calculated to depict relative changes from baseline during each tilt angle. Significance was determined using a one‐sided Student's *t*‐test. The threshold of significance was set at *p* < 0.05.

A Pearson correlation coefficient (*r*) was calculated using the mean delta values of all sheep and defined as follows: a correlation coefficient of 0–0.19 was considered very weak, 0.2–0.39 weak, 0.40–0.59 moderate, 0.6–0.79 strong, and 0.8–1 very strong. Correlation analysis and graph design were performed with GraphPad Prism v. 8.0.0.

### 2.7 Calculation of jugular venous flow

To calculate jugular venous blood flow (*F*) from velocity (*v*) and the cross‐sectional area (a) of the JV, the following formula was used:

F=v*a

### 2.8 Euthanasia

At the end of the trial, the sheep were euthanized under deep general anesthesia using pentobarbital (Eskonarkon®ad.us.vet., Streubli Pharma AG; 75 mg/kg BM) administered intravenously.

## 3 RESULTS

### 3.1 Baseline measurements

Mean baseline values of all sheep are summarized in Table 1.

**TABLE 1:** Summary of baseline values for assessed parameters

Area (cm^2^) JV	Width (cm) JV	Height (cm) JV	JV blood flow velocity (cm/s)	JV blood flow (ml/s)	JVP (mmHg)
0.81 ± 0.34	1.12 ± 0.40	0.87 ± 0.25	7.69 ± 5.32	6.03 ± 5.50	7.24 ± 1.44

ABP (mmHg)	CVP (mmHg)	HR (bpm)	ICP (mmHg)	ITP (mmHg)	CPP (mmHg)
80.51 ± 15.41	7.39 ± 4.80	93 ± 14	16.54 ± 10.10	18.12 ± 8.40	63.97 ± 15.33

Abbreviations: ABP, arterial blood pressure; CPP, cerebral perfusion pressure; CVP, central venous pressure; HR, heart rate; ICP, intracranial pressure; ITP, intrathecal pressure; JV, common jugular vein.

### 3.2 The venous system

#### 3.2.1 Cross‐sectional area of the common jugular vein

**FIGURE 2:**
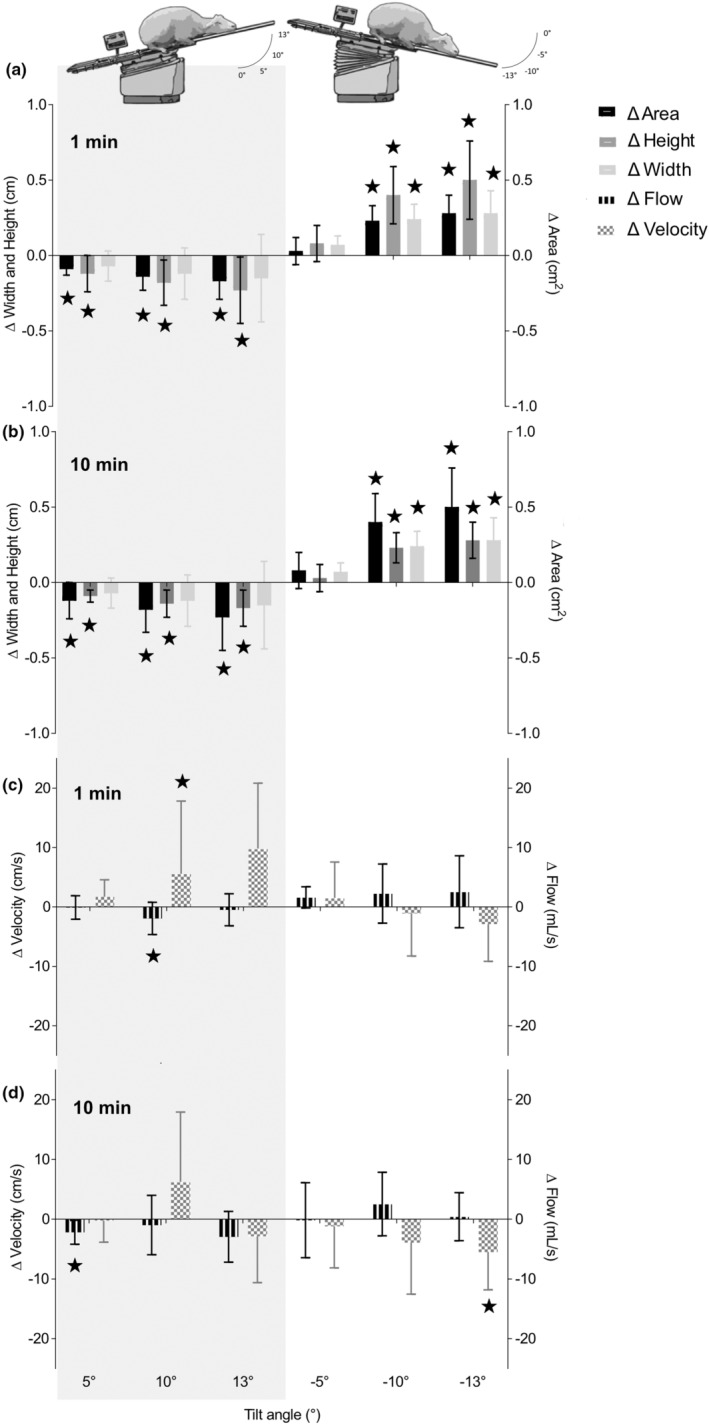
Postural‐induced changes in venous dynamics. (a) Relative changes in cross‐sectional area, width, and height of the common jugular vein during postural changes of head over body (HoB; +5°, +10°, +13°) and body over head (BoH; −5°, −10°, −13°) during minute 1 after the postural change. (b) Relative changes in cross‐sectional area, width, and height of the common jugular vein during postural changes of HoB (+5°, +10°, +13°) and BoH (−5°, −10°, −13°) during minute 10 after postural change. (c) Relative changes in common jugular vein blood flow and blood flow velocity during postural changes of HoB (+5°, +10°, +13°) and BoH (−5°, −10°, −13°) during minute 1 after the postural change. (d) Relative changes in common jugular vein blood flow and blood flow velocity during postural changes of HoB (+5°, +10°, +13°) and BoH (−5°, −10°, −13°) during minute 10 after postural change. Significant changes (p < 0.05) are indicated with an asterisk (★).

During the first minute of HoB tilting, the cross‐sectional area of the JV decreased significantly from baseline with each tilt angle (+5° (−0.13 ± 0.06 cm, *p* = 0.002), +10° (− 0.26 ± 0.17 cm, *p* = 0.006), +13° (−0.28 ± 0.24 cm, *p* = 0.017)), whereas it increased significantly at angles −10° (0.28 ± 0.26 cm, *p* = 0.021) and −13° (0.54 ± 0.27 cm, *p* = 0.002) during BoH tilting (Figure 2a). After 10 min of tilting, the area was still decreased significantly during the HoB tilt (+5° (−0.12 ± 0.12 cm, *p* = 0.029), +10° (−0.18 ± 0.15 cm, *p* = 0.016), +13° (−0.23 ± 0.22 cm, *p* = 0.026)), and remained significantly increased during the BoH tilt at −10° and −13° (0.4 ± 0.19 cm, *p* = 0.021, 0.5 ± 0.26 cm, *p* = 0.002, respectively; Figure 2b). There was a significant decrease in height of the JV during HoB tilting (−0.11 ± 0.03 cm, *p* < 0.001 (+5°), −0.18 ± 0.1 cm, *p* = 0.003 (+10°), −0.19 ± 0.11 cm, *p* = 0.005 (+13°)) while changes in width were marked but not significant during the first minute of each tilt angle (Figure 2a). During BoH, the height of the JV increased significantly at −10° and −13° (0.12 ± 0.08 cm, *p* = 0.007 and 0.14 ± 0.05 cm, *p* < 0.001, respectively; Figure 2a) and remained significantly increased at tilt angles −10° and −13° at 10 min (0.23 ± 0.1 cm, p = 0.001 and 0.28 ± 0.12 cm, *p* < 0.001, respectively; Figure 2b).

#### 3.2.2 Changes in jugular venous blood flow and velocity

HoB position: During the first minute of the HoB posture, mean blood flow in the JV was reduced non‐significantly at +5° (−0.1 ± 1.98 ml/s), +10° (−1.92 ± 2.71 ml/s) and + 13° (−0.47 ± 2.69 ml/s) angle with a more pronounced reaction at +10° than +13° (Figure 2c). At the +5° angle, a significant reduction in flow (−2.18 ± 2 ml/s, *p* = 0.022) was observed after 10 min (Figure 2d). In some animals, a slight increase in flow was seen at +10° and + 13° after 10 min (Figure 2d). JV blood flow velocity increased significantly during HoB tilting in the first minute at an angle of +13° (9.84 ± 11 cm/s, *p* = 0.039; Figure 2c). After 10 min, no significant difference in blood flow velocity compared with baseline was observed, and a decrease in velocity at +13° (−2.83 ± 7.78 cm/s) was recorded (Figure 2d).

BoH position: During the first minute of BoH posture, mean blood flow in the JV increased significantly at −5° (1.61 ± 1.78 ml/s, *p* = 0.038) when compared with baseline (Figure 2c). A marked increase was also observed at ‐10° (2.25 ± 4.97 ml/s) and ‐13° (2.55 ± 6.06 ml/s); however, due to the high inter‐individual variability, mean changes were not significant (Figure 2d). After 10 min, blood flow returned to baseline at −5° and −13°; however, at −10°, flow remained increased (2.54 ± 5.31 ml/s), but not significantly. Jugular venous blood flow velocity hovered around baseline at −5° (1.52 ± 6.04 cm/s) and −10° (−1.09 ± 7.16 cm/s), decreasing non‐significantly only at −13° (−2.89 ± 6.27 cm/s) (Figure 2d). Blood flow velocity decreased steadily at minute 10 of the BoH tilt, displaying a significant difference from baseline at −13° (−5.49 ± 6.33 cm/s, *p* = 0.043) (Figure 2d).

#### 3.2.3 Central venous pressure

During the HoB tilt, CVP remained steady at around baseline level, (+5°: 0.16 ± 1.51 mmHg (1 min), −0.21 ± 1.43 mmHg (10 min); +10°: 0.08 ± 2.66 mmHg (1 min), −0.16 ± 2.57 mmHg (10 min); +13°: −0.23 ± 3.55 mmHg (1 min), −0.31 ± 3.64 mmHg (10 min)) and decreased minimally and non‐significantly during the BoH tilt (−5°: 0.02 ± 1.07 mmHg (1 min), −0.17 ± 1.16 mmHg (10 min); −10°: −0.39 ± 0.99 mmHg (1 min), −0.48 ± 1.27 mmHg (10 min); −13°: −0.28 ± 1.1 mmHg (1 min), −0.51 ± 1.15 mmHg (10 min)). This was true for both, the first and last minute of the tilt test (Figure 4a,b).

**FIGURE 3:**
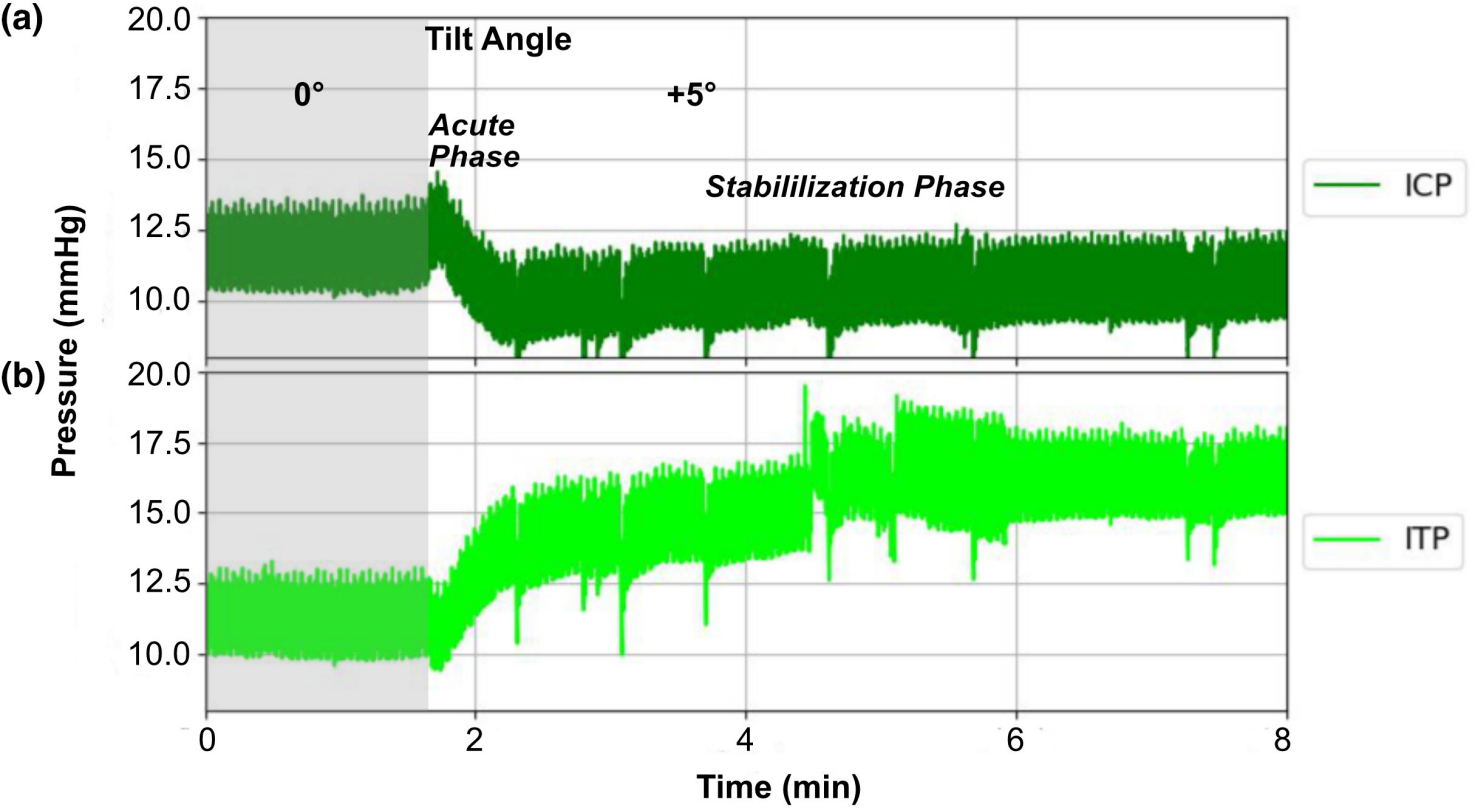
Representative Graph of biphasic changes in ICP during tilting from 0° to +5°. (a) Intracranial pressure (ICP) shown over 8 minutes depicting an acute phase and a stabilization phase. (b) Intrathecal Pressure (ITP) shown over 8 minutes, depicting an acute phase followed by a slower continuous increase (missing stabilization phase).

#### 3.2.4 Jugular venous pressure

**FIGURE 4:**
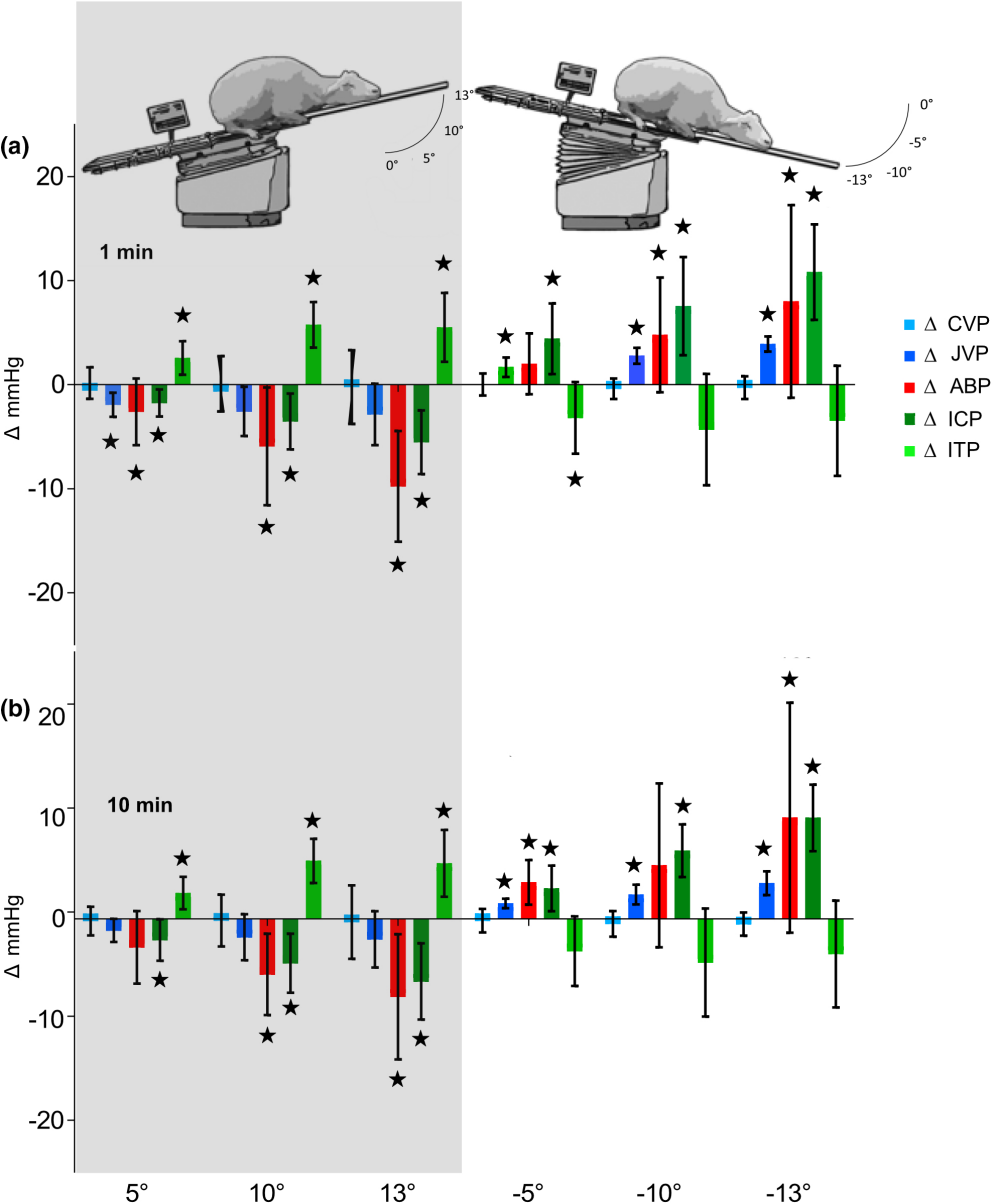
Postural‐induced pressure changes. (a) Relative changes to intracranial pressure (ICP), intrathecal pressure (ITP), central venous pressure (CVP), jugular venous pressure (JVP) and arterial blood pressure (ABP) during postural changes of head over body (HoB; +5°, +10°, +13°) and body over head (BoH; ‐5°, ‐10°, ‐13°) during minute one after postural change. (b) Relative changes to ICP, ITP, CVP, JVP and ABP during postural changes of head over body (HoB; +5°, +10°, +13°) and body over head (BoH; ‐5°, ‐10°, ‐13°) during minute 10 after postural change. Significant changes (*p* < 0.05) are indicated with an asterisk (★).

JVP decreased significantly from baseline at the +5° angle (−1.93 ± 1.16 mmHg, *p* = 0.022) during HoB and continued to decrease markedly but not significantly at the angles of +10° (−2.57 ± 2.36 mmHg, *p* = 0.059) and + 13° (−2.86 ± 2.96, *p* = 0.075; Figure 4a). After 10 min, JVP was still decreased, although non‐significantly, at each HoB tilt angle (+5°: −1.14 ± 1.14 mmHg, *p =* 0.069; +10°: −1.81 ± 2.28 mmHg, *p =* 0.105; +13°: −2.02 ± 2.78 mmHg, *p* = 0.120; Figure 4b). During BoH tilting, JVP increased significantly at each tilt angle (−5°: 1.69 ± 0.94 mmHg, *p* = 0.018; −10°: 2.78 ± 0.76 mmHg, *p* = 0.003; −13°: 3.91 ± 0.72 mmHg, *p* < 0.001) during the first minute (Figure 4a) and remained significantly increased at all tilt angles after 10 min (−5°: 1.54 ± 0.47 mmHg, *p* = 0.004; −10°: 2.42 ± 0.97 mmHg, *p* = 0.008; −13°: 3.54 ± 1.18 mmHg, *p* = 0.005, Figure 4b).

#### 3.2.5 Correlations of changes in the venous system

A very strong negative correlation (*r* = ‐0.97) between the cross‐sectional area of the JV and the tilting angle was found during the first minute of tilting. At minute 10 of the tilting, the cross‐sectional area of the JV and the tilting angle remained very strongly negatively correlated (*r* = −0.97). A very strong negative correlation (*r* = −0.94) was also found between the tilt angle and jugular venous flow during the first minute of each tilt angle, and the negative correlation remained strong (*r* = −0.84) at minute 10 of the tilt. With the decreasing cross‐sectional area of the JV, blood flow velocity increased consecutively, exhibiting a strong negative correlation (*r* = −0.79) during the first minute of tilting. There was a weak correlation between the cross‐sectional area of the JV and velocity during minute 10 of tilting (*r* = −0.33). At the same time, a decrease in the cross‐sectional area of the JV was initially very strongly positively correlated (*r* = 0.92) with jugular venous blood flow; this correlation remained very strong over time (*r* = 0.84). A moderate to strong positive correlation between tilting and CVP was found at both time points (*r* = 0.50 and *r* = 0.66). The cross‐sectional area of the JV and JVP correlated very strongly positively throughout the whole tilt test (*r* = 0.97, *r* = 0.97), i.e., pressure in the JV increased, when the area of the vessel increased. Jugular venous blood flow and JVP correlated very strongly positively during tilting at minute 1 (*r* = 0.95) and very strongly positively at minute 10 (*r* = 0.81), meaning that both JVP and blood flow increased steadily during the tilt test. CVP and jugular venous blood flow showed only a weak correlation during tilting. All correlations are listed in Table 2.

**TABLE 2:** Correlation matrix for assessed parameters. The correlation coefficient was determined. A correlation coefficient of 0–0.19 was considered very weak, 0.2–0.39 as weak, 0.40–0.59 as moderate, 0.6–0.79 as strong, and 0.8–1 as a very strong correlation. Correlations are shown at 1 and 10 min.

	Tilt angle		JV area		JV flow velocity		JVP		ABP		ITP		ICP		CVP	
	1 min	10 min	1 min	10 min	1 min	10 min	1 min	10 min	1 min	10 min	1 min	10 min	1 min	10 min	1 min	10 min
JV flow	−0.94	−0.84	+0.92	+0.84	−0.79	−0.33	+0.95	+0.81					+0.92	+0.82	−0.61	−0.59
JV Area	−0.97	−0.97			−0.89	−0.67	+0.97	+0.97					+0.99	+0.99	−0.58	−0.82
ABP	−0.99	−0.99					+0.97	+0.99					+0.99	+0.99	−0.41	−0.65
HR	−0.75	−0.72					+0.75	+0.67	+0.74	+0.65						
CVP	+0.50	+0.66					−0.57	−0.57	−0.41	−0.47			−0.53	−0.54		
ICP	−0.99	−0.99					+0.99	+0.99	+0.99	+0.99	−0.94	−0.94			−0.53	−0.72
ITP	+0.97	+0.97											−0.94	−0.94		

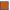
 very weak; 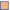 weak; 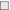 moderate; 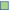 strong; 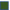 very strong.

### 3.3 Arterial blood pressure and heart rate

#### 3.3.1 Arterial blood pressure

While performing the HoB tilt, mean ABP showed a significant decrease from baseline at each tilting angle during the first minute (+5°: −2.6 ± 3.21 mmHg, *p* = 0.051; +10°: −5.93 ± 5.66 mmHg, *p* = 0.025 and + 13°: −9.77 ± 5.32 mmHg, *p* = 0.003; Figure 4a), which remained true for the last minute at +10° and + 13° (−5.50 ± 4.03 mmHg, *p* = 0.010 and −7.71 ± 6.21 mmHg, *p* = 0.014, respectively; Figure 4b). During the BoH tilt, ABP increased significantly from baseline at −10° (4.79 ± 5.51 mmHg, *p* = 0.043) and −13° (8.01 ± 9.26 mmHg, *p* = 0.04) during the first minute (Figure 4a), and at −5° (3.63 ± 2.22 mmHg, *p* = 0.005) and −13° (10.04 ± 11.39 mmHg, *p* = 0.044) during the last minute of the tilt angle (Figure 4b).

#### 3.3.2 Heart rate

During the tilting procedure, heart rate decreased minimally when performing the HoB tilt (mean delta over all HoB tilting angles at 1 min: −0.94 ± 4.4 bpm) and hovered around baseline while performing the BoH tilt (mean delta over all BoH tilting angles at 1 min: 0.49 ± 3.05 bpm). These findings pertain to the period during the first and last minute of tilting.

#### 3.3.3 Correlations of ABP, heart rate, and tilting angle

A very strong negative correlation was found between changes in mean ABP and tilt angle at 1 and 10 min after reaching the respective tilt angle (*r* = −0.99). A strong negative correlation was found between heart rate and tilt angle at 1 and 10 min (*r* = −0.75, *r* = −0.72, respectively). A strong positive correlation was found between heart rate and mean ABP (*r* = 0.74, *r* = 0.65). Correlations are summarized in Table 2.

### 3.4 CSF pressures

#### 3.4.1 Intracranial pressure

ICP decreased in a bi‐phasic manner with an initial drop followed by a stabilization phase (Figure 3a). The decrease was progressive with each tilting angle during the HoB tilt, exhibiting a significant difference from baseline at 1 min (+5°: ‐1.76 ± 1.30 mmHg, *p* = 0.010; +10°: −3.53 ± 2.68 mmHg, *p* = 0.011; +13°: −5.53 ± 3.06 mmHg, *p* = 0.003; Figure 4a) and at 10 min (+5°: −2.09 ± 2.05 mmHg, *p* = 0.027; +10°: −4.39 ± 2.92 mmHg, *p* = 0.007; +13°: −6.19 ± 3.78 mmHg, *p* = 0.005; Figure 4b). During BoH tilt, ICP increased significantly from baseline values with each decreasing tilting angle, which remained true for the first (−5°: 4.42 ± 3.39 mmHg, *p* = 0.012; −10°: 7.55 ± 4.72 mmHg, *p* = 0.006; −13°: 10.83 ± 4.59 mmHg, *p* = 0.001; Figure 4a) and last minute of tilting (−5°: 3.03 ± 2.26 mmHg, *p* = 0.011; −10°: 6.76 ± 2.61 mmHg, *p* < 0.001; −13°: 10.02 ± 3.3 mmHg, *p* < 0.001; Figure 4b).

#### 3.4.2 Intrathecal pressure

During HoB tilting ITP increased progressively with each tilting angle, however, after an initial fast increase, ITP continued to rise without a clear stabilization phase (Figure 3b). ITP displayed a significant difference from the baseline at each angle during the first and last minute of tilting (+5° (2.56 ± 1.61 mmHg, *p* = 0.005 and 3.03 ± 2.04 mmHg, *p* = 0.007), +10° (5.75 ± 2.19 mmHg, *p* < 0.001 and 5.67 ± 2.82 mmHg, *p* = 0.002), +13° (5.51 ± 3.32 mmHg, *p* = 0.005 and 4.51 ± 3.27 mmHg, *p* = 0.009); Figure 4a,b). When tilting the animal BoH during minute 1, ITP decreased significantly at first (−5°: −3.18 ± 3.45 mmHg, *p* = 0.036) but remained steady during the −10° (−4.32 ± 5.36 mmHg, *p* = 0.052) and −13° (−3.47 ± 5.29 mmHg, *p* = 0.085) steps (Figure 4a). After 10 min of BoH tilting, ITP was decreased non‐significantly from baseline level at angles −5° (−1.09 ± 1.72 mmHg, *p* = 0.091), −10° (−2.27 ± 3.4 mmHg, *p =* 0.081) and the −13° angle (−1.42 ± 3.85 mmHg, *p =* 0.205; Figure 4b).

#### 3.4.3 Correlations of CSF pressures

ICP and ITP correlated very strongly negatively with each other during the first (*r* = −0.94) and last (*r* = −0.94) minute during tilting, i.e., an increased ICP was followed by a decrease in ITP and vice versa. A very strong negative correlation can be found between ICP and tilt angle at both time points (*r* = −0.99). ITP, on the contrary, correlated very strongly positively with the tilting angle (*r* = 0.97) at 1 and 10 min of tilting. Correlations are summarized in Table 2.

### 3.5 Correlations of the vascular system and CSF pressures

A correlation between ICP and JVP was shown, with a very strong positive correlation of *r* = 0.99 during the first minute and *r* = 0.99 at minute 10. ICP and JV blood flow correlated very strongly positively (*r* = 0.92) during the first minute of tilting, mitigating a strong positive correlation during minute 10 (*r* = 0.82). ICP and CVP, in contrast, were only moderately negatively correlated (*r* = −0.53, *r* = −0.54). ICP correlated very strongly positively with JV cross‐sectional area (*r* = 0.99, *r* = 0.99). ICP and mean ABP furthermore correlated very strongly positively (*r* = 0.99) during the first minute and *r* = 0.99 during the last minute of tilting. Correlations are summarized in Table 2.

## 4 DISCUSSION

Despite the popularity of sheep as suitable large animals with which to model human neurological disorders (Murray & Mitchell, 2022; Trimmel et al., 2022), anatomical differences between human and sheep in the formation of the jugular vein might hamper the translatability of results of CSF dynamics studies. Effects of postural changes on ICP in humans are well described (Andresen et al., 2015; Andresen et al., 2016; Durward et al., 1983; Magnaes, 1976a, 1976b; Magnaes, 1978; Norager et al., 2020; Qvarlander et al., 2013), with the postural transition from a supine to an upright position resulting in a biphasic decrease of ICP characterized by an acute decrease, followed by a stabilization phase (Gergele & Manet, 2021). Postural changes in ICP are thereby thought to be governed by CSF transfer from the cranial to the spinal compartment (Klarica et al., 2014; Magnaes, 1976a). A craniocaudal direction of CSF movement with a subsequent narrowing of the cervical subarachnoid space and widening of the epidural veins in that region was previously observed using MRI examination in patients during changes of body position from horizontal to upright (Alperin, Hushek et al., 2005; Alperin, Lee et al., 2005). Detailed studies in anesthetized cats and models thereof have thereby shown for the described narrowing of the cervical arachnoid space to cause a “disconnection” of the cranial CSF column from the spinal CSF column in the 90° upright position. Thus, the pressure in the lumbar subarachnoid space corresponded directly to the hydrostatic height of the CSF column between the foramen magnum and the site of lumbar cannulation, while the intracranial pressure gradient resulted from the hydrostatic height between the site of measurement, i.e. the lateral ventricle, and the foramen magnum (Klarica et al., 2014). In humans, the biphasic behavior is explained by venous hydrostatic pressures and the collapse particularly of the internal jugular vein effectively preventing the ICP from falling below −15 mmHg in an upright position (Chapman et al., 1990; Holmlund et al., 2018). The posture‐induced collapse of the internal jugular vein is considered a passive process; thus, to maintain CPP in the upright position, the CSF and venous systems are thought to be complemented by the active regulatory effects of the arterial system (Liu et al., 2020; Matakas et al., 1975; Panerai et al., 2019).

Postural‐induced decrease in ICP is also described in a variety of quadruped animals, such as rodents, cats, and dogs (Kotani, Nitta, et al., 1992, Eftekhari et al., 2019, Sturges et al., 2019). However, little is known about the comparability of postural ICP regulation by the venous and arterial systems in quadrupedal compared with bipedal species.

In sheep, as in other ruminants, the common jugular vein is the largest vein in the neck and drains most blood from the head and the neck (Lu et al., 2008; Hoffmann et al., 2014). In the present study, sheep were subjected to passive postural changes ranging from 0° to +13° HoB and from 0° to −13° BoH, during table tilt testing under general anesthesia. The chosen angles were considered physiological, as sheep are often subjected to these postural changes in alpine terrain. HoB tilting of the sheep led to an acute decrease in ICP and an increase in ITP. Changes in ICP were thereby directly correlating to the tilt angle. The onset of the decrease of ICP was immediate, with a lack of progression over the 10 min during which the tilt angle was maintained. A comparable stabilization phase, as described in humans, occurred within the first minute following postural change. The decrease in ICP was further accompanied by a reduction in the cross‐sectional area of the JV with respective changes in venous blood flow (decreased) and blood flow velocity (increased). JVP was decreased markedly possibly due to a hydrostatic venous blood shift from the head toward the heart. The relatively small changes in CVP observed in our sheep at the +13° angle might be attributed to the rather high vascular compliance of veins; changes in volume shifted by the +13° angle appeared to lead to only relatively small changes in CVP (Tansey et al., 2019). Postural‐induced changes in CVP in humans have been described previously (Amoroso & Greenwood, 1989; Tansey et al., 2019) and are described to correlate with hydrostatic load in the HoB posture in anesthetized monkeys (Terada & Takeuchi, 1993). In the same study, gravitational blood shift during BoH, in contrast to HoB, appeared to be impeded directly by the central tendon of the diaphragm causing non‐symmetrical changes in CVP when comparing HoB with BoH posture in monkeys (Terada & Takeuchi, 1993), which may explain the relatively small changes in CVP measured in the anterior vena cava in our sheep study. To gain a more integrated understanding of the underlying mechanism on posture‐induced CVP changes in sheep, venous pressure in the posterior vena cava, caudal to the diaphragm, should also be measured. All sheep were ventilated mechanically throughout the experiment. Mechanical ventilation has shown to affect CVP in humans by increasing intra‐thoracic pressure (Shojaee et al., 2017). CVP magnitude changes appear, however, to be associated more directly with an increase in positive end‐expiratory pressure settings independently of posture (Hong et al., 2009).

In the BoH position, jugular venous pooling can be suspected due to the marked increase in JVP and cross‐sectional area of the JV. Venous blood pooling has previously been described in the BoH posture in giraffes (Brondum et al., 2009). BoH was further associated with a significant drop in ABP in giraffes, most likely due to baro‐ and volume receptors in the carotid artery causing a precapillary vascoconstriction (Brondum et al., 2009). In our sheep study, a significant increase in ABP was measured.

Systemic ABP is known to fluctuate only minimally with postural changes due to autoregulatory mechanisms (Currens, 1948). During HoB tilting, a significant decrease in mean carotid ABP and heart rate was recorded in our sheep. Orthostatic hypotension was described previously in anesthetized rabbits as a dysfunction of the baroreflex system during anesthesia (Willeford, 2011). Acute gravitational stress is thereby thought to pool venous blood in the lower half of the body, thus transiently reducing venous backflow to the heart and consequently reducing cardiac output.

One can only speculate that an anesthesia‐induced dysfunction of baro‐ and volume receptors occurs also in this setting, which might be a major limitation of this study. Volatile anesthetics, as used in this setting, are known to suppress venous baroreflex, mean arterial pressure, heart rate, and sympathetic efferent outflow (McCallum et al., 1994). To confirm our preliminary findings and conclude our assessment, survival chronic long‐term studies in awake sheep are currently being conducted to gain further insights into CSF dynamics in non‐anesthetized sheep and their translatability to humans.

## 5 CONCLUSION

Despite quadrupedalism and known anatomical differences between humans and sheep, in particular in the formation of the JV, sheep showed widely comparable biphasic effects of postural changes on ICP during HoB and BoH tilting. A marked collapse of the JV was observed during HoB, in accordance with findings in humans. Active regulatory effects of the arterial system possibly on maintaining CPP and cerebral blood flow were observed independently of tilting direction. This study underlines the suitability of sheep as a translatable model with which to study CSF dynamics.

## AUTHOR CONTRIBUTIONS

Miriam Weisskopf, Marianne Schmid Daners, Margarete Arras, Anthony Podgoršak, Simone Jucker, Nina Eva Trimmel, and Markus Florian Oertel created the research plan and design. Miriam Weisskopf, Nina Eva Trimmel, and Simone Jucker performed animal preparation, instrumentalization, monitoring, and euthanasia. Anthony Podgoršak, Marianne Schmid Daners, Nina Eva Trimmel, Miriam Weisskopf, and Simone Jucker carried out data collection. Anthony Podgoršak oversaw data post‐processing. Markus Florian Oertel conducted the neurosurgical procedures. Nina Eva Trimmel and Miriam Weisskopf analyzed the data and wrote the manuscript. The final version of this manuscript was revised and approved by all authors.

## FUNDING INFORMATION

This study was supported by the Swiss National Science Foundation (SNSF) through grant no. 315230_184913 / 1, as well as by the Olga Mayenfisch Foundation and the Hartmann‐Müller Foundation.

## CONFLICT OF INTEREST

The authors declare no competing interest.

## ETHICAL STATEMENT

Animal housing and all experimental procedures were approved by the local Committee for Experimental Animal Research (Cantonal Veterinary Office Zurich, Switzerland) under the license number ZH119/2019 in conformity with the European Directive 2010/63/EU of the European Parliament and the Council on the Protection of Animals used for Scientific Purposes, and the Guide for the Care and Use of Laboratory Animals procedure (European Parliament, 2010).

## Data Availability

The datasets used and/or analyzed during the current study are available from the corresponding author upon reasonable request.
